# Abdominal Hypertension after Abdominal Plication in Postbariatric Patients: The Consequence in the Postoperative Recovery

**DOI:** 10.1055/s-0043-1772587

**Published:** 2023-11-30

**Authors:** Martin Morales-Olivera, Erik Hanson-Viana, Armando Rodríguez-Segura, Marco A. Rendón-Medina

**Affiliations:** 1 Department of Plastic and Reconstructive Surgery in Hospital General de Tláhuac, Mexico City, Mexico; 2 Department of Plastic and Reconstructive Surgery in Hospital General de la Ciudad de México “Dr. Rúben Leñero,” Mexico City, Mexico

**Keywords:** postbariatric surgery, intra-abdominal pressure, postoperative recovery, abdominal plicature, abdominal hypertension

## Abstract

**Background** Abdominoplasty with abdominal plication increases intra-abdominal pressure (IAP) and has been previously associated with limited diaphragmatic excursion and respiratory dysfunctions. Many factors found in abdominoplasties and among postbariatric patients predispose them to a higher occurrence. This study aims to evaluate the impact of abdominal plication among postbariatric patients, assess whether the plication increases their IAP, and analyze how these IAP correlate to their postoperative outcome.

**Methods** This prospective study was performed on all patients who underwent circumferential Fleur-De-Lis abdominoplasty. For this intended study, the IAP was measured by an intravesical minimally invasive approach in three stages: after the initiation of general anesthesia, after a 10-cm abdominal wall plication and skin closure, and 24 hours after the procedure.

**Results** We included 46 patients, of which 41 were female and 5 were male. Before the bariatric procedure, these patients had an average maximum weight of 121.4 kg and an average maximum body mass index of 45.78 kg/m ^2^ ; 7 were grade I obese patients, 10 were grade II, and 29 were grade III. Only three patients were operated on with a gastric sleeve and 43 with gastric bypass. We presented six patients with transitory intra-abdominal hypertension in the first 24 hours, all of them from the grade I obesity group, the highest presented was 14.3 mm Hg. We presented 15% (7/46) of complication rates, which were only four seroma and five dehiscence; two patients presented both seroma and wound dehiscence.

**Conclusion** Performing a 10-cm abdominal wall plication or greater represents a higher risk for intra-abdominal hypertension, slower general recovery, and possibly higher complication rate in patients who presented a lower degree of obesity (grade I) at the moment of the bariatric surgery.

## Introduction

Kron et al 1 were the first to successfully associate intra-abdominal pressure (IAP) to pathophysiological changes, describing the intra-abdominal compartment syndrome. IAP can rise for different reasons and cause tissue damage when it is severe. When muscle plication is performed in the abdominoplasty, it is well known that the IAP may rise. Yet, the risks and the physiological changes they may carry are still inconclusive. 2 Patient safety and medical error reduction are crucial in every elective cosmetic surgery. One of the most severe complications in elective plastic surgery is venous thromboembolism (VTE), ranging from a subclinical presentation to a severe pulmonary embolism (PE), leading to significant morbidity and death. Any procedure can cause VTE. However, many factors found in abdominoplasties and among postbariatric patients predispose them to higher complications; one of the most common factors is long operative time, higher bleeding, low postoperative mobilization, and higher IAP.

Abdominoplasty, in conjunction with abdominal plication, is an aesthetic surgical intervention that defines the waist contour by tightening the abdominal wall and removing excess skin and fat—often used in postpregnancy patients or massive weight loss patients, where diastasis recti are prominent. These maneuvers increase intra-abdominal tension and have been previously associated with respiratory dysfunctions and limiting diaphragmatic excursion. 3

Previous studies have shown an improvement in life quality and weight control among postbariatric patients after body contouring surgery. 4 The abdominoplasty is the most required procedure among these patients. 5 However, these patients have particular tissue characteristics that make them prone to complications. So, this study aims to evaluate the impact of abdominal plication among postbariatric patients, assess whether the plication increases their IAP, and analyze how these IAP correlate to their postoperative outcome. To our knowledge, no previous study has focused on abdominal plication among postbariatric patients with Fleur-De-Lis procedure, nor the clinical implications of an elevation of IAP in the recovery of these patients amid patient mobility and independence.

## Methods

This prospective study was performed in “Hospital General de Tlahuac.” Approval of the study protocol was obtained from the institutional ethics committee, and all patients gave written informed consent before the operation. Inclusion criteria were patients with a weight reduction of at least 75% of their excess weight after a gastric bypass or gastric sleeve, aged between 25 and 60 years old. Exclusion criteria for this study were patient refusal, history of smoking, obstructive sleep apnea, and body mass index (BMI) below 18.5 or above 35 kg/m ^2^ at the moment of the plastic surgery reconstruction. In addition, patients with cardiac dysfunction, respiratory problems, and renal and hepatic dysfunctions were excluded from the study. Preoperatively, transoperative, and postoperative essential information was summarized in Microsoft Excel 2016 (Microsoft Corp., Redmond, WA).

The IAP was recorded in all patients who underwent circumferential Fleur-De-Lis abdominoplasty from February 2019 to June 2021. For this intended study, the IAP was measured by an intravesical minimally invasive approach. 6 7 Following induction of general anesthesia, a standard Foley catheter was inserted, and the bladder was emptied. After the bladder was insured empty, 75 mL of sterile saline was instilled into the empty bladder, and the catheter tubing was clamped distal to the aspiration port. We used 75 mL since it has been reported and validated that at 50 to 100 mL, the bladder acts as a passive diaphragm and can accurately reflect IAP in normal conditions and under general anesthesia. 8 9 Through the aspiration port of the catheter, a Stryker compartmental pressure monitor (Stryker-Leibinger, Kalamazoo, MI) was used to measure the intravesical pressure at end-expiration. Again, the symphysis pubis was maintained as the zero-reference point. Three measurements were taken, following the same steps: first after initiation of the general anesthesia, the second after abdominal wall plication and skin closure, and the third, taken 24 hours postoperatively.

**Fig. 1:**
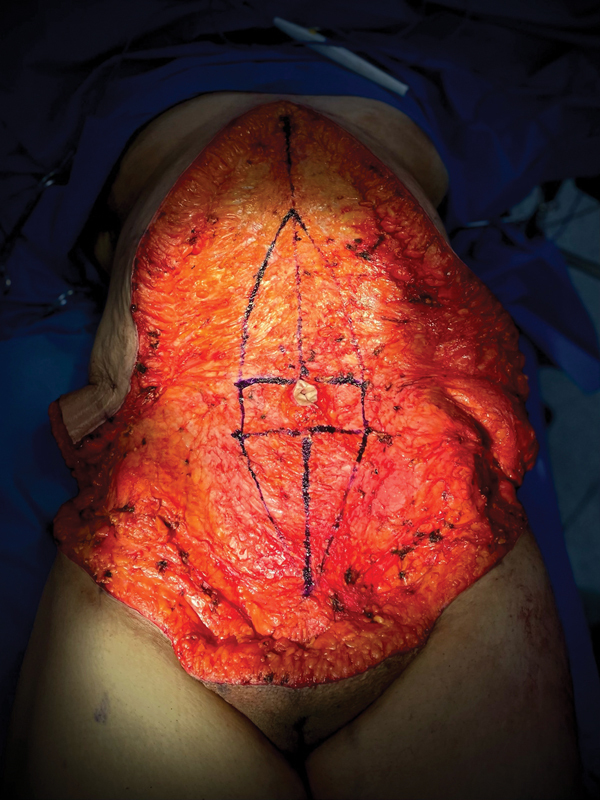
A 10-cm abdominal wall plication was made in the postbariatric patient during the circumferential Fleur-De-Lis abdominoplasty.

After total flap elevation, a midline vertical plication of the rectus muscle and myofascial components was performed. A 10-cm plication was marked at 3 cm superior and inferior to the umbilicus, and the plication gap was closed until reaching the xiphoid and pubic region ( Fig. 1 ). None of the patients presented a plication of 2 cm further than the medial rectus abdominal border. The plication was made with an interrupted “X” plication suture 10 with a 0 polypropylene (Prolene, Ethicon US, LLC, Somerville, NJ), followed by a continuous suture with a 2–0 barbed polyglyconate (V-Loc, Covidien, Mansfield, MA), from the xiphoid and pubis to the umbilicus. After this, the adipocutaneous flap was sutured to the fascia and closed according to the patient's characteristics, leaving two ¼-inch closed drains, one on the back and one on the front. The foley catheter was withdrawn after 24 hours postoperative (after the last IAP measurement). Compression garments were used in all patients until the third postoperative day. The Barthel activity index (BAI) was obtained from the first postoperative day to the 10th postoperative day, and their relationship with IAP was compared.

### Statistical Analysis

Statistics are presented as means standard deviation. The *t* -test was used for statistical analysis of continuous data and confirmed by an appropriate nonparametric test (Mann–Whitney or Wilcoxon matched pairs). Independent relationships will be made with chi-square to determine the degrees of freedom between the different variables independently and assess their statistical significance. The level *p* = 0.05 was considered as the cutoff value for significance.

## Results

We included 46 patients, of which 41 (90.5%) were female and 5 were male; the average age was 42.9 years (28–59). Before the bariatric procedure, these patients had an average maximum weight of 121.4 kg (81.9–145.3, ± 22.13) and an average maximum BMI of 45.78 kg/m ^2^ (31.2–58.7, ± 8.67), 7 (15.2%) were grade I obese patients, 10 (21.7%) grade II, and 29 (63.1%) grade III. The average weight loss after the bariatric procedure was 51.6 kg, corresponding to an average of 92% excess weight decrease. The BMI at the moment of the plastic surgery reconstruction was of 26.68 kg/m ^2^ (20.5–33.2, ± 3.18). Only three patients were operated on with a gastric sleeve and 43 with gastric bypass; the average time till the Fleur-De-Lis procedure was 29.8 months after the bariatric surgery. Diabetes mellitus type 2 was the most common comorbidity, with a prevalence of 17%, followed by hypertension at 9%. All the patients underwent a Fleur-De-Lis procedure, with an average flap resection of 5.2 kg (± 2.3), presented with a degree of diastasis of 8.9 cm (± 1.3), a surgical time of 242 minutes, and bleeding of 485 mL. Only one patient required a blood transfusion and had transoperative bleeding of 1,200 mL. The mean IAP before surgery was on average of 2.6 mm Hg, in all groups there was a significant increase transoperatively ( *p* ≤ 0.001). However, only the obesity I group, presented an increase in the next 24 hours after surgery, in contrast to the other groups that presented a significant decrease ( *p* ≤ 0.02). This previous was correlatable to the BAI; the higher the IAP postoperatively, the lower the BAI score ( *p* ≤ 0.001). The rest of the information is summarized in Table 1 .

**Table 1:** The preoperative, transoperative, and postoperative results of the intra-abdominal pressure and the Barthel activity index among the prebariatric obesity groups

Obesity	No.	IAP in mm Hg					BAI				
		Pre op	Trans op	*p* -Value	24 h	*p-* Value	Score	Mild	Moderate	Severe	*p* -Value
I	7	3.6	10.7	<0.001	12.8	<0.001	34.3	0 (0)	1 (14)	6 (86)	<0.001
II	10	2.9	9.7	<0.001	8.6	0.02	68.1	7 (70)	3 (30)	0 (0)	<0.001
III	29	2.2	7.7	<0.001	6.3	0.001	69.8	23 (79)	6 (21)	0 (0)	<0.001
Total	46	2.6	8.7	<0.001	8.0	0.01	62.9	30 (65)	10 (21)	6 (13)	<0.001

Abbreviations: BAI, Barthel activity index; IAH, intra-abdominal hypertension; Preop, preoperative; Transop, transoperative.

Note: Values are presented as numbers (%).

Most patients had 1 day of hospital stay, except for seven patients (15.2%) that had only 2 days. The reasons for the extra day of stay were secondary to abdominal discomfort in three patients, postoperative nausea in three patients, and transfusion requirements in one. We presented 15% (7/46) of complication rates, which were only two seromas and three dehiscence; two patients presented both seroma and wound dehiscence. The complication rate was not found to be associated with the presence of age ( *p* = 0.42), sex ( *p* = 0.31), diabetes ( *p* = 0.22), weight loss after the bariatric surgery ( *p* = 0.67), tissue excised ( *p* = 0.38), time of operation ( *p* = 0.46), bleeding ( *p* = 0.09). However, in only one patient blood transfuse was necessary after surgery, that patient later on presented a dehiscence. The average tissue excised among the patients that did not present any complication was 5.231 kg (1.9–10.5, ± 2.24 kg/m ^2^ ), which was similar to the patients that presented complications 5.233 kg (1.5–9.1, ± 3.09 kg/m ^2^ ), no statistical significance was found ( *p* = 0.38). Two of the six patients that presented intra-abdominal hypertension (IAH; 12–15 mm Hg) presented complications. However, no strong correlation was found ( *p* = 0.18). On the other hand, we found that a severe BAI score was correlated with a higher complication rate ( *p* ≤ 0.001). The rest of the complications compared with the BAI are summarized in Table 2 and against IAH is presented in Table 3 .

**Table 2:** Results of complication in relationship with the Barthel activity index

BAI	No.		Complications			*p* -Value
		Seroma	Dehiscence	Seroma + dehiscence	Total	
Mild	30	0	1	1	2 (6.6)	0.27
Moderate	10	1	0	0	1 (10)	0.60
Severe	6	1	2	1	4 (33)	<0.001
Total	46	2	3	2	7 (15)	<0.001

Abbreviation: BAI, Barthel activity index.

Note: Values are presented as numbers (%).

**Table 3:** Results of complication in relationship with intra-abdominal hypertension

IAH	No.	Complications				*p* -Value
		Seroma	Dehiscence	Seroma + dehiscence	Total	
No	40	2	2	1	5 (12.5)	0.50
Grade I (12–15 mm Hg)	6	0	1	1	2 (33.3)	0.18
Grade II (16–20 mm Hg)	0	0	0		0 (0)	–
Total	46	2	3	2	7 (15.2)	–

Abbreviation: IAH, intra-abdominal hypertension.

Note: Values are presented as numbers (%).

## Discussion

Bariatric surgery has increased by more than 900% from 1990 to 2008 and continues to increase in recent years, resulting in more contouring surgeries among these patients. 11 So, it is seldom to understand the physiological changes presented in the contour surgeries among these patients.

In previous studies, the IAP has been shown to increase for two reasons in abdominoplasties: the plication of the abdominal wall (horizontal force), followed by the adipocutaneus flap resection and skin closure (vertical force). 12 13 Increased IAP has been linked with decreased perfusion in the abdominal wall and its adjuvants tissues, such as skin and fatty tissue, due to a direct compression effect in major vessels, directly impacting the epigastric arteries. 14 This further can reduce wound healing, making these patients prone to wound dehiscence, necrosis, infection, or seroma, among others. However, this has not been proven statistically significant, probably due to small samples. In our study, we found a higher incidence of seroma and dehiscence among patients that presented with IAH (>12 mm Hg); nonetheless, it was not significant ( *p* = 0.18). In addition, several studies have associated dyspnea with the degree of abdominal plicature. However, this tends to be mild to moderate, manageable with supplementary oxygen and respiratory exercises and resolved shortly. 2 15 16 17 Abdominal compartment syndrome is sporadic, and only four cases have been reported in the literature. 18 19 Besides from respiratory implications of abdominal plicature, 2 3 13 15 and its relationship with a hospital stay, 20 no previous study has focused on a postoperative activity or abdominal plication among massive weight loss patients.

Our study found that when patients had grade II and III obesity at the time of the bariatric surgery, the IAP after the abdominal plication and the Fleur-De-Lis abdominoplasty tended to decrease rapidly in the first 24 hours postoperatively ( *p* ≤ 0.001). And on the contrary, it was found to increase among the grade I obesity group in the first 24 hours ( *p* ≤ 0.001), even reaching an IAH grade I in six out of the seven patients included in this group, without presenting any systemic complications nor symptoms beside abdominal discomfort. One may think that this is a result of a higher stretched abdominal wall before their bariatric surgery, since with a higher grade of obesity achieved, the more significant visceral fat is present and abdominal fascia stretching, leading to a more adaptive relaxation after plicature tension. This may be primarily because of the histologic anomalies found among postbariatric gastric bypass patients, like disarray of dermal elastic fibers, thickened and poorly organized collagen fibers, and fewer and less active fibroblasts, collapsed adiposities with a cytoplasmic membrane thickening, among others. 21 22 23

Because, during the procedure, none of the patients presented with an excessive clinical tightening of the abdominal wall nor any surgical difficulty, and most of the time, a tighter plication could be achieved in all groups. None of the patients presented a plication of 2 cm further than the medial rectus abdominal border. Also, taking notice that among postbariatric patients, it is commonly seen after a few moments that the abdominal wall appears loose again, giving an impression of a lack of adequate plication. This was previously emphasized a couple of months after surgery, finding a much looser skin and even an abdominal wall bulge in some patients.

A direct impact of IAP and general recovery ( *p* ≤ 0.001) was also noticed in this study. However, we could not find a statistical significance in rates of complications among patients with IAH ( *p* = 0.18), probably due to a small patient sample. Although a relationship was found among patients with a severe BAI and a higher complication rate ( *p* ≤ 0.001), where only grade I obese patients presented this (6/7). This previous meaning that higher mobility and independence to perform their functions was found in grade II and III obesity groups. The 46 patients achieved independence (according to the BAI) on the seventh day after the Fleur-De-Lis abdominoplasty. In our patients, we did not find any cases of VTE or systemic repercussions.

Nevertheless, one of VTE's main protective factors is postoperative mobility and ambulation. Something that is well achieved in patients that presented a lower postoperative IAP ( *p* < 0.001). We could not demonstrate this in this study since more patients are needed to demonstrate this relationship. Still, a decrease in daily functions and mobility in the early postoperative may increase the possibility of more severe complications like VTE and PE.

Several limitations were met in our study, first, more patients were needed to find more statistical significance results, as well as the measurements of other indirect alterations secondary to IAP increase, that could be associated with wound healing problems, such as skin perfusion, respiratory capacity, and oxygen saturation, among others. Also, we lacked IAP measurement after abdominal wall plication and previous skin closure. However, we thought that the IAP increase will not be as significant in our type of setting, compared with a 29 to 31% increase found in conventional abdominoplasties and nonpostbariatric patients. 12 13 This is because, in Fleur-De-Lis abdominoplasty patients are not bent at the waist to facilitate skin closure, plus their multiple and long skin incisions help distribute tension.

In our experience, we've found that greater plications of 10 cm, even though technically easy, will not drastically affect the end aesthetic result of the Fleur-De-Lis abdominoplasty; on the contrary, it may increase the postoperative risks and higher postoperative pain.

Following this, in our practice, we routinely do a 10-cm abdominal plication in patients with grade II and III obesity at the time of the bariatric surgery, without reaching more than 2 cm further than the rectus abdominal muscles, and in patients with a prebariatric obesity grade I, we prefer to do a more conservative abdominal plication, even though a bigger plication could easily be achieved. And if a 10-cm plication is opted by the surgeon on these previous patients, an IAP lesser than 10 mm Hg should be ensured with intravesical measurements at the moment of skin closure.

Performing a 10-cm abdominal wall plication in the circumferential Fleur-De-Lis abdominoplasty in massive weight loss patients is effective and safe. However, it represents a higher risk for IAH, slower general recovery, and possibly higher complication rate in patients who presented a lower degree of obesity (grade I) at the moment of the bariatric surgery. Probably due to lower abdominal tissue compliance, as found in grades II to III. Furthermore, a more extensive study is needed to associate the grade of plication to more severe and systemic complications like VTE and PE among massive weight loss patients.
